# Zero-exemplar deep continual learning for crop disease recognition: a study of total variation attention regularization in vision transformers

**DOI:** 10.3389/fpls.2023.1283055

**Published:** 2024-01-04

**Authors:** Boyuan Wang

**Affiliations:** School of Computer Science and Engineering, Faculty of Innovation Engineering, Macau University of Science and Technology, Macao, Macao SAR, China

**Keywords:** smart agriculture, plant disease detection, deep learning, continual learning, vision transformer

## Abstract

With the increasing integration of AI technology in the food industry, deep learning has demonstrated its immense potential in the domain of plant disease image recognition. However, there remains a gap in research between models capable of continual learning of new diseases and addressing the inherent catastrophic forgetting issue in neural networks. This study aims to comprehensively evaluate various learning strategies based on advanced computer vision models for multi-disease continual learning tasks in food disease recognition. To cater to the benchmark dataset requirements, we collected the PlantDiseaseCL dataset, sourced from the internet, encompassing diverse crop diseases from apples, corn, and more. Utilizing the Vision Transformer (ViT) model, we established a plant disease image recognition classifier, which, in joint learning, outperformed several comparative CNN architectures in accuracy (0.9538), precision (0.9532), recall (0.9528), and F1 score (0.9560). To further harness the potential of ViT in food disease defect recognition, we introduced a mathematical paradigm for crop disease recognition continual learning. For the first time, we proposed a novel ViT-TV architecture in the multi-disease image recognition scenario, incorporating a Total Variation (TV) distance-based loss (TV-Loss) to quantify the disparity between current and previous attention distributions, fostering attention consistency and mitigating the catastrophic forgetting inherent in ViT without prior task samples. In the incremental learning of the PlantDiseaseCL dataset across 3-Steps and 5-Steps, our strategy achieved average accuracies of 0.7077 and 0.5661, respectively, surpassing all compared Zero-Exemplar Approaches like LUCIR, SI, MAS, and even outperforming exemplar-based strategies like EEIL and ICaRL. In conclusion, the ViT-TV approach offers robust support for the long-term intelligent development of the agricultural and food industry, especially showcasing significant applicability in continual learning for crop disease image recognition.

## 1 Introduction

Plant diseases reduce the yield and quality of food, leading to significant economic losses and reducing food safety at the national and global levels (Savary et al., 2019). Plant disease surveillance is critical for preventing disease spread (Jones, 2021; Ristaino et al., 2021). However, current monitoring approaches rely on regular field identification by agroforestry specialists or farmers’ knowledge of plant diseases. This manual inspection-based technique is time-consuming and costly, and it also necessitates specialists’ a high level of field knowledge. Therefore, the development of smart agriculture requires a detection system that can automatically identify the type of plant disease and the exact location of the lesion.

With the advancement of AI technologies, researchers have utilized machine learning and image processing techniques to develop systems to automatically detect plant diseases such as apple disease(Chakraborty et al., 2021), wheat disease (Nema and Dixit, 2018), cotton disease (Bhimte and Thool, 2018), and corn disease (Kusumo et al., 2018). Color, shape, and texture information are used to construct feature vectors, which are then classified using random forest (Mekha and Teeyasuksaet, 2021), support vector machines (SVM) (Banerjee and Madhumathy, 2022), etc. However, traditional machine learning and image processing methods rely heavily on prior knowledge and require human design based on disease characteristics, making it difficult to use big data to discover feature patterns automatically (Liu and Wang, 2021). The essence of these techniques is by manually designing features and developing classifiers (or rules) and using computer image processing methods such as image segmentation methods (Prewitt, Sobel), feature extraction methods (SIFT, HOG) and classification methods (SVM). When the environment or the type of plant disease changes, it is always required to change the threshold or redesign the algorithm, which is inefficient for detection in real and complex natural environments (Liu and Wang, 2021). Therefore, the classification performance is low, the model lacks stability, and the adaptability is poor.

After the emergence of deep learning technology, an important branch of AI, models for end-to-end disease detection by learning features from different fields, scenarios, and scales have become a research hotspot in the field of smart agriculture and food industry. Deep learning techniques can automatically learn features from massive amounts of data and cope with specific complex changes in the natural environment (Boulent et al., 2019; Liu and Wang, 2021). Models for plant disease image recognition based on deep learning techniques belong to deep neural networks (DNN), including the classic convolutional neural network (CNN) (Albawi et al., 2017) and the latest ViT (Dosovitskiy et al., 2020), etc. The CNN architectures include VGG16 (Simonyan and Zisserman, 2015), ResNet (He et al., 2016), NASNet (Zoph et al., 2018), Inception V3 (Szegedy et al., 2016), MobileNet (Howard et al., 2017), EfficientNet (Tan and Le, 2019), etc. All these models are very deep neural networks formed by stacking multiple convolutional layers. All above models have been applied to the study of plant disease identification.

In reference to Sultana et al. ‘s study (Habiba and Islam, 2021), they utilized the VGG16 model for identifying diseased tomatoes through transfer learning. The study focused on ten different categories of tomato leaf images from the Plant Village dataset, namely: a) Target Spot, b) Yellow leaf, c) Mosaic Virus, d) Bacterial Spot, e) Early Blight, f) Leaf Mold, g) Late Blight, h) Septoria Leaf spot, i) Spider Mites, and j) Healthy Leaf. The dataset consists of a varying number of photos per class, ranging between 1500 and 3000. To ensure proper training, validation, and testing, the dataset was divided into 60% for training data, 20% for validation data, and 20% for test data. The results showed satisfactory classification performance with an accuracy of about 95.5%. Brahmaji et al. (Godi et al., 2022a) used the ResNet-152 V2 model for automatic disease identification on a tomato leaf image dataset containing ten different diseases. The processing flow designed mainly consisted of Pre-processing of leaf structure, leaf feature extraction, leaf analysis and segmentation, and leaf classification process. After training, The ResNet-152 V2 model achieved 95% detection accuracy. Yang et al. (Yang et al., 2020) developed a plant disease image classification model based on NASNet’s extended neural network and attention mechanism. Their study used a dataset consisting of 58,200 crop leaf images, including 37 different classes of healthy/diseased crops. The results show that the fine-grained NASNet Large neural network model based on the attention mechanism achieves excellent classification performance with 95.62% accuracy, which is well suited for automatically detecting crop diseases. Haque et al. (Haque et al., 2022) collected 5939 images of maize crops from experimental fields located in three maize growing areas, including three types of diseases: Maydis leaf blight, Turcicum leaf blight, and Banded leaf and sheath blight, as well as healthy ones. They used the basic architecture of the advanced CNN model “Inception-v3” network to build three models on the maize dataset, viz. flatten layer with fully connected layer (Inception-V3_flatten-FC), global average pooling layer (Inception-v3_GAP) and global average pooling layer with fully connected layer (Inception-V3_GAP-FC). Of these, Inception-v3_GAP achieved the highest accuracy of 95.99% in a separate test set and was efficient in learning relevant features of the disease and predicting the correct category in unseen data. Rajbongshi et al. (Rajbongshi et al., 2020) used the MobileNet model with a transfer learning approach to detect rose plant diseases on an image dataset of powdery mildew, black spot, rust, and dieback diseases. They used 1600 data images to train the model and 400 data images to test the model. As a result, the MobileNet model with the transfer learning method obtained an accuracy of about 95.63%. Vijayalata et al. (Vijayalata et al., 2022) focused their research on identifying four diseases affecting cassava yield: Cassava Bacterial Blight, Cassava Brown Streak Disease, Cassava Mosaic Disease, and Cassava Green Mottle. They used the EfficientNet-B0 model for the early detection of these diseases. A total of 21,367 cassava images comprised the original image dataset, which was divided into 20 test cases and 80% of the training data, and 20% of the validation data. An accuracy of 92.6% was achieved after the model was applied to the test cases.

Zhuang (Zhuang, 2021) suggested a ViT model-based method for identifying viral diseases in cassava leaf images. The image dataset of cassava leaves was provided by Makerere Artificial Intelligence Lab in a Kaggle competition, including four subtypes of diseases and healthy cassava leaves. After applying the K-Fold cross-validation method, their model achieved a classification accuracy of 90.02% on the test set. Zhang et al. (Zhang et al., 2021) proposed a new rice disease recognition method based on the Swin Transformer architecture (a new variant of ViT), including sliding window operation and hierarchical design. The proposed model was trained with images of five rice diseases (bacterial blight, rice blast, rice false smut, brown spot, and sheath blight) in the field environment and achieved a classification accuracy of 93.4% on the test set, which is about 4.1% higher than that of traditional machine learning models. Li et al. (Li and Li, 2022) proposed a lightweight ViT-based disease detection model, ConvViT, for apple disease identification in complex environments. ConvViT includes a convolutional structure and a Transformer structure, and the detection accuracy result (96.85%) is comparable to the performance of the current state-of-the-art Swin-Tiny. The parameters and FLOPs are only 32.7% and 21.7% of Swin-Tiny, significantly ahead of CNN models such as MobilenetV3 and Efficientnet-b0.

Both CNN and Transformer architectures have demonstrated exceptional capabilities in detecting plant diseases, surpassing the expertise of agroforestry professionals in certain tasks. Nonetheless, the majority of these models are anchored in static datasets and unchanging settings, overlooking the fact that information often unfolds progressively. As a result, they struggle to assimilate and adapt to fresh insights. On occasion, they might completely break down or exhibit pronounced deterioration in tasks they once mastered, culminating in profound issues of catastrophic forgetting (Hadsell et al., 2020). This phenomenon, where neural networks lose prior knowledge, was first pinpointed by McCloskey and Cohen in 1989 (McCloskey and Cohen, 1989). When juxtaposed with these artificial models, the human aptitude for learning is rooted in a diverse array of neurocognitive processes and brain memory systems. Such complexities underpin our ability to hone skills and embed memories for the long haul, as detailed by German I. Parisi et al. in 2019 (Parisi et al., 2019).

Drawing from the principles of cognitive science, the realm of continual learning, as articulated by Lesort et al. (Lesort et al., 2020) endeavors to confront the aforementioned limitations in artificial intelligence. To achieve a balance between preserving old knowledge and learning new knowledge, continual learning algorithms face a trade-off known as the stability-plasticity dilemma (Abraham and Robins, 2005; Wu et al., 2021; Araujo et al., 2022). In the traditional static learning, data follows independent and identically distributed (IID) distributions, where data is sampled according to the same probability distribution. In typical IID data sets D , we have D∼P(x,y) , where P(x,y) denotes the joint probability distribution of the data generation. However, in a continual learning environment, the data probability distribution of the data set D is no longer a typical IID probability distribution but is instead divided into several distinct subsets $D_{t}$ . Let $D=\cup_{t=1}^{T}D_{t}$ , where each subset represents a single task and is sampled from T different IID probability distributions $P_{t}{\left(x,y\right)}_{t=1}^{T}$ . In a continual learning environment, the dataset D can be represented as $D=\cup_{t=1}^{T}D_{t}$ with $D_{t}∼P_{t}\left(x,y\right)$ , where $D_{t}$ represents the subset corresponding to the t -th task and $P_{t}\left(x,y\right)$ represents the probability distribution of the t -th task. The characteristic of continual learning is that it learns from dynamic data distributions, allowing for more flexible and adaptive machine learning systems.

At present, the food industry sees limited exploration and utilization of Continual Learning techniques in AI-driven smart solutions. To bridge this gap, we undertook this investigation, outlining our primary findings below.

The potential of the ViT model in the food industry, particularly in food disease defect recognition, necessitates its enhancement and evaluation for continual learning capabilities. Establishing advanced continual learning visual models in the domain of food and crop disease prevention is imperative. Addressing these challenges, this study was undertaken, and the following key contributions were made:

1. To meet the benchmark dataset requirements for multi-disease continual learning classification tasks, we curated the PlantDiseaseCL dataset from the internet, encompassing diverse food diseases from apples, corn, and more. Using the ViT-S/16 model, we developed a food disease image recognition classifier. In joint learning evaluations, the ViT-S/16 outperformed several other CNN architectures in metrics such as accuracy, precision, recall, and F1 score.
2. Beyond just model performance, we delved into the model’s feature learning capability using the t-SNE method. Visualization of feature vectors learned by different models revealed that the ViT-S/16 demonstrated superior classification outcomes in feature distribution, excelling in inter-class separability in feature embeddings.
3. To maximize the potential of ViT in food disease defect recognition, we introduced a mathematical paradigm for continual learning of crop disease defects. We proposed the novel ViT-TV architecture for multi-disease image recognition, incorporating a Total Variation distance (Rudin et al., 1992; Bhojanapalli et al., 2021) loss (TV-Loss) to quantify disparities between current and previous attention distributions.
4. By optimizing the overall loss function of ViT with TV-Loss and Cross-Entropy Loss, we balanced model stability and plasticity, maintaining attention consistency during the learning process of new and old tasks, thereby mitigating the catastrophic forgetting inherent in ViT without the need for storing samples from previous tasks. This offers a new attention alignment method for ViT in multi-disease continual learning scenarios.
5. To validate our proposed ViT-TV, we designed 3-stage and 5-stage continual learning processes on the PlantDiseaseCL dataset. We assessed various continual learning methods from perspectives such as attention alignment, global importance parameter regularization, and knowledge transfer between teacher-student networks for new and old tasks.
6. Further comparisons of different attention alignment method variants were made, juxtaposing our ViT-TV with the original ViT and other methods like Jensen–Shannon divergence, Hellinger distance, and Bhattacharyya distance, thereby confirming the efficacy of our approach.

These contributions establish a novel framework for continual learning in image classification tasks for food disease recognition. The proposed Zero-Exemplar approach ViT-TV method fosters advancements in multi-disease recognition technology, enhancing the model’s capability to continuously learn new diseases, and underpinning the long-term intelligent evolution of the food industry.

The remainder of this paper is organized as follows. The sources and construction methods for training, verifying, and testing datasets are described in Section II. Section III describes our proposed approach, ViT-TV and performance evaluation metrics. Model parameter settings for the experimental study are discussed in Section IV. The experimental results and discussion are presented in Section V. Section VI conclude the paper with comments on future work.

## 2 Materials and methods

### 2.1 Datasets

To validate our proposed methodology, we collected the PlantDiseaseCL dataset, specifically designed for continual learning evaluations. This dataset comprises 30,863 disease images of various foods, including apples, corn, pepper, and potatoes, all of which were collected from the Internet. Each image is standardized to a resolution of 256 × 256 pixels. For structured evaluation, the dataset has been segmented into training, validation, and testing subsets, detailed further in **Table 1**. For the broader research community’s benefit, we have made the PlantDiseaseCL dataset publicly available on the Kaggle platform. It can be accessed at https://www.kaggle.com/datasets/gabrielwang01/leaf-disease-must (last accessed on 18 August 2023).

**Table 1:** Training, validation, and test sets for the PlantDiseaseCL dataset.

Class	Images	Dataset		
		Training	Validation & Testing	Total
Apple healthy	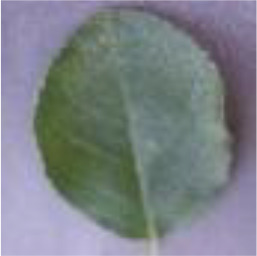	1506	1004	2510
Apple Black Rot	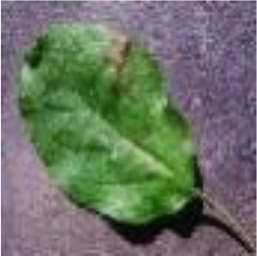	1490	994	2484
Apple rust	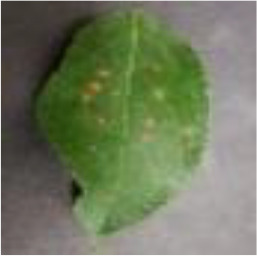	1320	880	2200
Apple scab	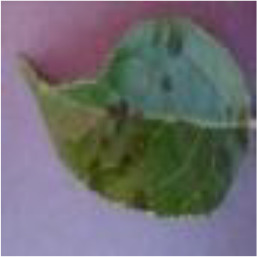	1512	1008	2520
Corn healthy	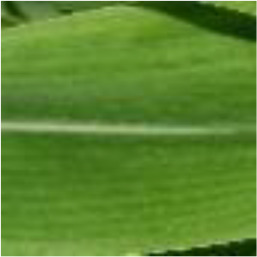	1394	930	2324
Corn common rust	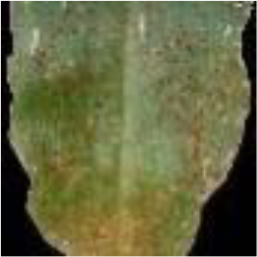	1430	954	2384
Corn gray leaf spot	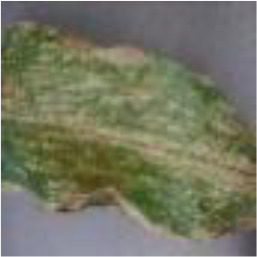	1232	820	2052
Corn northern leaf blight	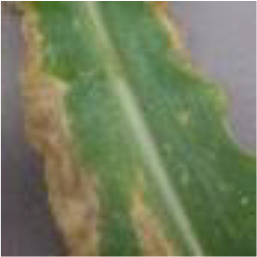	1431	954	2385
Pepper healthy	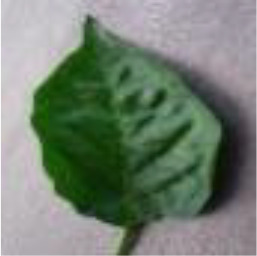	1491	994	2485
Pepper bacterial spot	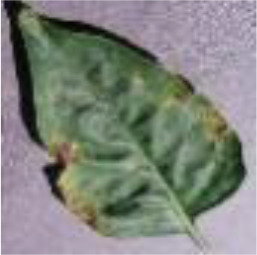	1435	956	2391
Potato healthy	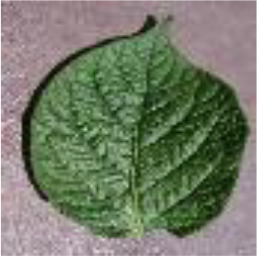	1368	912	2280
Potato early blight	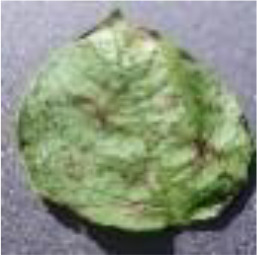	1454	970	2424
Potato late blight	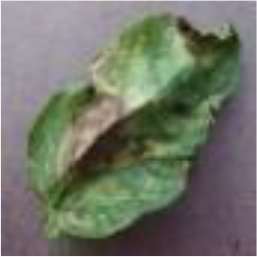	1454	970	2424
Total		18517	12346	30863

### 2.2 Methods

#### 2.2.1 Multi-disease continual learning paradigm for crops and foods

Continual learning for multi-disease detection in crops plays a significant role in improving agricultural productivity. The ability to detect, differentiate, and act upon a growing variety of diseases over time can significantly impact the crop yield and the overall food supply chain. The mathematical paradigm outlined for multi-disease medical image recognition can similarly be adapted to design a theoretical framework for crops.

Let’s define our dataset for crop disease recognition as $C=\{\left(x_{j},z_{j}\right){\}}_{\left\{j=1\right\}}^{m}$ , where $x_{j}$ denotes the sample feature (e.g., an image of a plant or crop’s leaf, hyperspectral data) within the feature space X and $z_{j}$ is the corresponding label within the label space Z . Here, (X) represents the input space detailing the features (or symptoms) exhibited by crops due to diseases or other external factors. In contrast, Z is the output space indicating the type of crop disease or health status.

**Incremental Learning over Time**: In real-world scenarios, new crop diseases may emerge, or previously studied diseases might show new symptoms. Therefore, similar to the multi-disease medical paradigm, our dataset C will be split into K stages, each containing $m_{k}$ data points, where $m_{k}\in M$ and $\sum_{k=1}^{K}m_{k}=m$ . The parameter set or vector M serves as a means to control the distribution of data points across stages, allowing for flexibility and adaptation based on specific requirements.

**The Learning Objective**: For the crop multi-disease scenario, at each stage, our goal is to generate a model that can recognize all the diseases observed so far without forgetting the previously learned diseases. Mathematically, the objective at the k+1  stage is expressed as shown in Equation 1.


(1) $$\begin{array}{c} \theta_{\text{k}+1}=\arg\underset{\text{θ}}{\min}L\left(g_{\text{k}+1}\left(\cdot ,\theta_{\text{k}}\right),C_{\text{k}+1}\right) \end{array}$$

where $g_{k+1}$ represents the model at the k+1  stage, θ is the parameter set or vector of the model, and $C_{k+1}=C_{k+1}^{'}\cup^{}\left\{\left(x,z\right)\in C:x\in X_{k}\right\}$ is the combined dataset of the prior k+1 tasks and the specific crop disease at task k.

**Dealing with Catastrophic Forgetting**: To avoid catastrophic forgetting, we need to integrate techniques such as Regularization-based Approach, which adds a penalty to the loss function to ensure the weight changes for a new task do not drastically deviate from the learned weights for previous tasks.

Given: Original loss function: ℒ , Weights of the neural network: W , Previously learned weights: $W_{prev}$ ,

The new loss function, incorporating the regularization term, can be expressed as shown in Equation 2.


(2) $$\begin{array}{c} {ℒ}_{new}=ℒ+\lambda \sum_{i}{\left(W_{i}-W_{prev,i}\right)}^{2} \end{array}$$

where λ is a regularization parameter. The summation is taken over all weights in the network. The continual learning approach, when correctly applied to crops, can lead to proactive disease management, better yields, and a more resilient food system. The mathematical paradigm above sets the foundation for building AI systems that can evolve with changing disease landscapes in agriculture.

#### 2.2.2 ViT-TV: aligning ViT attention using total variation distance

In our study, we enhanced the original ViT model (Dosovitskiy et al., 2020) to address the challenges of continual learning across multiple diseases. The core concept behind Vision Transformers (ViT) revolves around processing based on image patches.

**For the original ViT**: Given an image I of dimensions (H×W×C) , where (H) and (W) represent the height and width of the image, and C denotes the number of channels, we partition the image into n  patches, each of size (P×P) . Thus, $n=\frac{H\times W}{P\times P}$ .

Each image patch  i can be linearly embedded into a vector $v_{i}\;$ of dimension D , as shown in Equation 3.


(3) $$\begin{array}{c} v_{i}=MA\left[patch_{i}\right] \end{array}$$

where  MA is an embedding matrix with dimensions D×(P×P×C) . In this context, $patch_{i}$ serves as an index to select a specific row from the embedding matrix MA . These embeddings are then processed through L Transformer layers. Each Transformer layer consists of two primary components: Multi-Head Self-Attention (MHSA) and a Multi-Layer Perceptron (MLP). The design of MHSA aims to capture information in parallel across different representational subspaces. Briefly describing its operation, this structure first projects the input data into multiple representational spaces, each having its unique set of queries, keys, and values.

For h  heads, each head has its distinct set of projection matrices: $\left\{P_{i}^{Q},P_{i}^{K},P_{i}^{V}\right\}$ , where i  denotes the $i^{th}$ head. These matrices project the original input data into their respective subspaces, as shown in Equation 4.


(4) $$\begin{array}{c} Q_{i}=\text{Input}\cdot P_{i}^{Q},\;K_{i}=\text{Input}\cdot P_{i}^{K},\;V_{i}=\text{Input}\cdot P_{i}^{V} \end{array}$$

In their respective subspaces, for each head i , a standard attention operation is executed, as illustrated in Equation 5.


(5) $$\begin{array}{c} A_{i}=\text{Softmax}\left(\frac{Q_{i}K_{i}^{T}}{\sqrt{d_{i}}}\right)V_{i} \end{array}$$

Where $d_{i}$ represents the dimension of the $i^{th}$ head. Finally, the outputs from all heads are concatenated and passed through a shared output transformation, resulting in the final outcome, as depicted in Equation 6.


(6) $$\begin{array}{c} \text{Output}=\text{Concat}\left(A_{1},\ldots ,A_{h}\right)\cdot P^{O} \end{array}$$

Where $\left(P^{O}\right)$ is the weight matrix of the output transformation. This multi-head structure enables the model to capture various features and dependencies in parallel across multiple subspaces, enhancing the model’s expressive capability.

**Global Total Variation Distance Regularization**: In continuous recognition of crop diseases, as time progresses, new diseases might emerge, or the manifestations of known diseases may evolve. Thus, we can represent the continuous disease recognition tasks as described in Equation 7.


(7) $$\begin{array}{c} \left[T_{disease1},T_{disease2},\ldots ,T_{diseasen}\right] \end{array}$$

For each disease task $\left(T_{disease\left(i\right)}\right)$ , there exists a unique data distribution: [*P(image, label T _disease(i)_*)], where image represents the image data of crop leaves, and denotes the disease label. Prior to Equation 9, the assumption is made that the covariance between two diseases is zero, denoted as $\Sigma_{disease\left(i\right),j}=0$ . This assumption implies that the parameter distributions of different diseases are statistically independent. Mathematically, it can be expressed as represented in Equation 8.


(8) $$\Sigma_{disease\left(i,j\right)}=E\left[\left(\text{θ}-{\text{μ}}_{disease\left(i\right)}\right){\left(\text{θ}-{\text{μ}}_{disease\left(j\right)}\right)}^{T}\right]=0$$

Here, $\Sigma_{disease\left(i,j\right)}$ represents the covariance matrix between disease $T_{disease\left(i\right)}$ and $T_{disease\left(j\right)}$ , where θ denotes the model parameters, and $\mu_{disease\left(i\right)}$ and $\mu_{disease\left(j\right)}$ represent the mean parameters for diseases $T_{disease\left(i\right)}$ and $T_{disease\left(j\right)}$ , respectively.

This assumption signifies that the learning of parameters for one disease does not influence the parameters of other diseases. By assuming independence between disease-specific parameter distributions, we establish a foundation for further derivation and utilization of Equation 9 in addressing continual learning tasks.

To learn on a specific disease, we typically aim to maximize the following likelihood function:


(9) $$\begin{array}{c} L\left(\text{θ}|T_{disease\left(i\right)}\right)=\sum_{\left(image,label\right)\in D_{disease\left(i\right)}}\log P\left(label|image;\theta ,T_{disease\left(i\right)}\right) \end{array}$$

Where $D_{disease\left(i\right)}$ is the dataset for disease $T_{disease\left(i\right)}$ and θ  represents the model parameters.

Probability Distribution Shift: In the continuous recognition tasks of crop diseases, as new diseases emerge or known disease manifestations change, the model needs to be updated continuously. Suppose the model parameter distribution after disease $\left(T_{disease\left(i\right)}\right)$ is $\left(P\left(\text{θ}|T_{disease\left(i\right)}\right)\right)$ . When encountering a new disease $\left(T_{disease\left(j\right)}\right)$ , we desire the model parameter distribution to be $\left(P\left(\text{θ}|T_{disease\left(j\right)}\right)\right)$ .

We employ Bayesian updating to describe this process, as expressed in Equation 10.


(10) $$\begin{array}{c} P\left(\text{θ}|T_{disease\left(j\right)},D_{disease\left(j\right)}\right)\propto P\left(D_{disease\left(j\right)}|\text{θ},T_{disease\left(j\right)}\right)P\left(\text{θ}|T_{disease\left(i\right)},D_{disease\left(i\right)}\right) \end{array}$$

Where $\left(P\left(\text{θ}|T_{disease\left(j\right)},D_{disease\left(j\right)}\right)\right)$ is the posterior distribution, representing the distribution of the model parameters (θ) given the new disease $\left(T_{disease\left(j\right)}\right)$ and its associated data $\left(D_{disease\left(j\right)}\right)$ . $\left(P\left(D_{disease\left(j\right)}|\text{θ},T_{disease\left(j\right)}\right)\right)$ is the likelihood function, indicating the probability of observing the data $\left(D_{disease\left(j\right)}\right)$ given the model parameters (θ) and the disease $\left(T_{disease\left(j\right)}\right)$ . $\left(P\left(\text{θ}|T_{disease\left(i\right)},D_{disease\left(i\right)}\right)\right)$ is the prior distribution, which describes our belief about the distribution of the model parameters (θ) before considering the disease $\left(T_{disease\left(i\right)}\right)$ and its data $\left(D_{disease\left(i\right)}\right)$ .

In continuous recognition tasks of crop diseases, maintaining knowledge from historical learning is crucial. Like other continual learning tasks, when introducing new disease categories or encountering new data distributions, we might face the risk of “catastrophic forgetting”, where the process of acquiring new knowledge might disrupt what has been previously learned.

To effectively address this issue, we introduced the TV distance (Rudin et al., 1992; Bhojanapalli et al., 2021) as a regularization technique for the first time in continuous recognition of crop diseases. The TV distance provides us with a means to evaluate the parameter changes in the model across continuous tasks.

The TV distance provides a measure to gauge the difference between two probability distributions associated with the parameters of neural networks. In the context of Bayesian, we treat the weights and biases of the neural network as random variables, effectively viewing the entire set of parameters as a probability distribution. Given two such distributions, $\left(p\left(\text{θ}|D_{disease}\right)\right)$ and $\left(q\left(\text{θ}|D_{disease}\right)\right)$ , which represent the distributions of the whole neural network parameters under two different disease conditions, the TV distance between them is defined as shown in Equation 11.


(11) $$\begin{array}{c} \text{TV(p,q)}=\frac{1}{2}\int |p\left(\theta |D_{disease}\right)-q\left(\theta |D_{disease}\right)|\text{d}\theta \end{array}$$

For discrete distributions, this formula can be written as expressed in Equation 12.


(12) $$\begin{array}{c} TV\left(p,q\right)=\frac{1}{2}\sum_{\text{θ}}\left|p\left(\text{θ}|D_{disease}\right)-q\left(\text{θ}|D_{disease}\right)\right| \end{array}$$

The core idea behind TV distance is to describe the maximum deviation of two probability distributions for the same event in the context of crop diseases. Considering the posterior distribution of parameters for old tasks $\left(q\left(\text{θ}|D_{disease\left(i\right)}\right)\right)$ and the posterior distribution of parameters based on new disease data $\left(p\left(\text{θ}|D_{disease\left(i\right)+1}\right)\right)$ , our optimization objective can be expressed as represented in Equation 13.


(13) $$\begin{array}{c} L_{T_{disease\left(i\right)+1}}\left(\text{θ}\right)=-\sum_{\left(x,y\right)\in D_{disease\left(i\right)+1}}\log P\left(yx;\theta ,T_{disease\left(i\right)+1}\right)\\ +\lambda TV\left(p\left(\text{θ}|D_{disease1:i+1}\right),q\left(\text{θ}|D_{disease1:i}\right)\right) \end{array}$$

Where:

- $D_{disease1:i}$ and $D_{disease1:i+1}$ represent the dataset up to disease i and the dataset up to disease i+1 , respectively.
- $p\left(\text{θ}|D_{disease1:i+1}\right)$ is the posterior distribution of the parameters θ based on the new disease data (up to disease i+1) .
- $\;q\left(\text{θ}|D_{disease1:i}\right)$ denotes the posterior distribution of the parameters  θ  based on the old disease data (up to disease i ).
- TV(p,q) stands for the Total Variation distance, which assesses the difference between two probability distributions  p  and  q  specific to crop diseases.
- θ encapsulates the model’s parameter set, defining its structure and behavior, and is typically adjusted during training to minimize the loss function.
- (x,y) is a sample pair, with x being the input (e.g., a crop image) and y  the corresponding label (e.g., disease category).
- λ is a regularization coefficient, determining the weight of the TV distance in the overall loss. Adjusting λ allows for a balance between the loss for task i+1 and the change in parameter distribution. Setting λ too high might render the model overly conservative, hindering adaptation to the new task, while a value too low might cause an overemphasis on the new task, risking the forgetting of old tasks.

**Figure 1:**
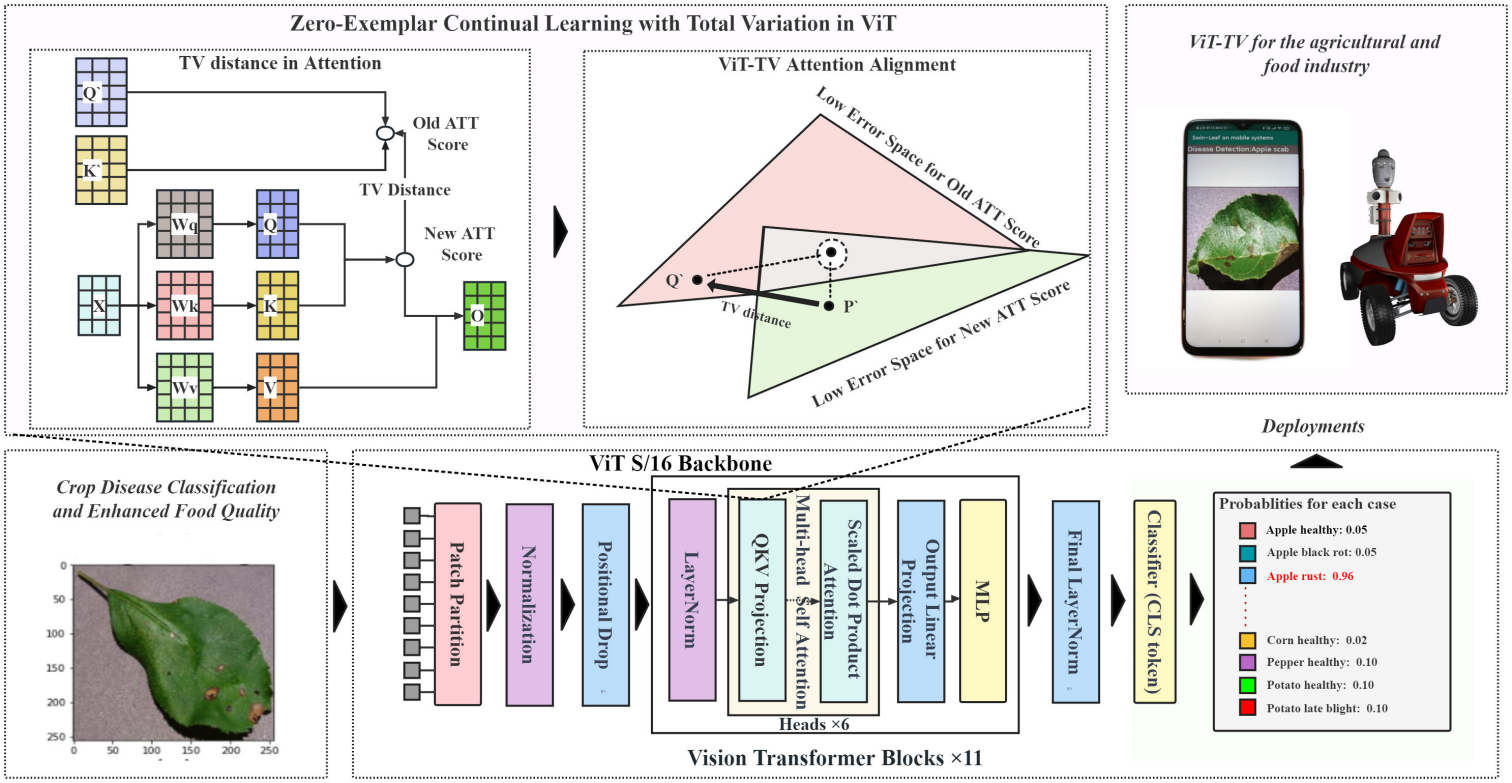
Framework for crop disease continual learning with ViT-TV attention alignment.


**ViT-TV Attention Alignment**: In this study, we introduce an enhanced model, termed ViT-TV, that aligns attention based on the TV distance, as depicted in **Figure 1**. The ViT model aims to integrate the continual learning recognition task of crop diseases by leveraging attention mechanisms. Given an input feature matrix (X) with dimensions B×N×C  , where  B denotes batch size, N represents sequence length, and C signifies feature dimensions, the model initially undergoes a linear transformation to obtain a combined representation for Query(Q) , Key(K) , and Value(V) , as shown in Equation 14:

(14) $$\begin{array}{c} QKV=XW_{qkv} \end{array}$$

Here, $W_{qkv}$ is a weight matrix. After this transformation, the combined representation QKV is reshaped and permuted to separate out the individual representations for Q , K , and V . Specifically, QKV is reshaped to dimensions $\left(B,N,3,H,\frac{C}{H}\right)$ , where B denotes the batch size, N represents the sequence length, and H stands for the number of attention heads. The tensor is then permuted to rearrange these dimensions, resulting in individual tensors for Q , K , and V . Subsequently, attention scores ATTN are computed, as illustrated in Equation 15.


(15) $$\begin{array}{c} ATTN=\left(Q\times K^{T}\right)\times \frac{1}{\sqrt{d}} \end{array}$$

Where (d) is the dimension size of each attention head, utilized to scale the dot product. For each score, the softmax function is applied to ensure the sum equals 1 across the last dimension, as demonstrated in Equation 16.


(16) $$\begin{array}{c} ATTN^{\prime }=\text{Softmax}\left(ATTN\right) \end{array}$$

The computed attention weights are then dot-multiplied with the Value matrix (V), as represented in Equation 17:


(17) $$\begin{array}{c} Z=ATTN^{\prime }\times V \end{array}$$

Finally, Z undergoes another linear transformation followed by a dropout layer to produce the model’s output. When training the model on a new crop disease recognition task, while ensuring it retains knowledge from previous tasks, we employ attention disparities to compute the regularization loss. Each attention matrix is reshaped from (B×h×w) to ((B×w)×h) , where h and w are the height and width of the attention matrix, respectively. To ensure the sum of weights in each attention matrix equals 1, normalization is applied, as represented in Equation 18.


(18) $$\begin{array}{c} P^{\prime }=\frac{\left|P\right|}{\sum_{j=1}^{h}P_{ij}},\;\;Q^{'}=\frac{\left|Q\right|}{\sum_{j=1}^{h}Q_{ij}} \end{array}$$

Where P denotes the attention matrix associated with the previous task, encapsulating the model’s attention distribution during that phase. Conversely, Q signifies the attention matrix pertinent to the current task, illustrating the model’s attention distribution for the new task at hand. The matrices $P^{\prime }\;$ and $Q^{\prime }$ represent their normalized counterparts, ensuring a standardized attention distribution across the matrix dimensions.

The absolute value operation ensures all values are positive, and normalization ensures the sum of each row equals 1. The difference between the two normalized attention matrices is then computed using the Total Variation distance, as demonstrated in Equation 19.


(19) $$\begin{array}{c} \text{TV}\left(P^{\prime },Q^{\prime }\right)=\frac{1}{2}\sum_{i=1}^{N}\sum_{j=1}^{h}\left|P_{ij}^{'}-Q_{ij}^{'}\right| \end{array}$$

For all attention matrices, the TV distances are accumulated to compute the overall loss, as indicated in Equation 20.


(20) $$\begin{array}{c} \text{Total\_Loss}=\sum_{i=1}^{len\left(\text{attention\_list}\right)}\text{TV}\left(P_{i}^{'},Q_{i}^{'}\right) \end{array}$$

To effectively balance the learning of the new crop disease recognition task and the retention of knowledge from previous tasks, we introduce a composite loss consisting of two components:

Cross-Entropy Loss: For the new crop disease recognition task, we compute the cross-entropy loss between the model's predictions and the actual labels, as expressed in Equation 21.


(21) $$\begin{array}{c} L_{\text{cross-entropy}}=-\sum_{i=1}^{N}y_{i}\log\left(\hat{y_{i}}\right)] \end{array}$$

Where $y_{i}$ is the actual label, and $\hat{y_{i}}$ is the model’s prediction.

Attention Regularization Loss: Based on the aforementioned description, we have computed the TV distance between two attention matrices, which serves as the regularization loss, as expressed in Equation 22.


(22) $$\begin{array}{c} L_{\text{attention}}=\text{TV\_Loss} \end{array}$$

This loss ensures that during training on a new crop disease recognition task, the model doesn’t drastically alter its attention weights from previous tasks. Ultimately, these two losses are combined into a total loss, where λ is a hyperparameter to balance the two, as shown in Equation 23.


(23) $$\begin{array}{c} L_{\text{total}}=L_{\text{cross-entropy}}+\lambda \times L_{\text{attention}} \end{array}$$

Our objective is to adjust the model parameters to minimize the TV distance, ensuring that predictions on new disease data closely align with the true distribution while maintaining consistency with old disease data. By optimizing this composite loss, the ViT-TV model can retain knowledge of previous tasks while learning new crop disease recognition tasks, even without sample replay.

#### 2.2.3 Evaluation metrics

Accuracy is the ratio of correctly predicted samples to the total number of samples, as represented in Equation 24.


(24) $$\begin{array}{c} Accuracy=\frac{True\;Positives+True\;Negatives}{True\;Positives+True\;Negatives+False\;Positives+False\;Negatives} \end{array}$$

Precision is the ratio of true positives to the sum of true positives and false positives, as expressed in Equation 25.


(25) $$\begin{array}{c} Precision=\frac{True\;Positives\;}{True\;Positives+False\;Positive}\left(25\right) \end{array}$$

Sensitivity is the ratio of true positives to the sum of true positives and false negatives, as shown in Equation 26.


(26) $$\begin{array}{c} Sensitivity=\frac{True\;Positives}{True\;Positives+False\;Negative} \end{array}$$

F1-score is a measure that combines precision and sensitivity into a single metric, as illustrated in Equation 27.


(27) $$\begin{array}{c} F1-score=\frac{2\times \left(Sensitivity\times Precision\right)}{Sensitivity+Precision} \end{array}$$

These metrics are used to evaluate the performance of classification models. Precision measures the proportion of true positive predictions among all positive predictions. Sensitivity measures the ability of the model to identify true positive samples. The F1-score provides a balanced assessment of precision and sensitivity. AUC provides a comprehensive evaluation of model performance across different thresholds.

To evaluate the CL capability of a model $M_{t}$ that has learned a set of tasks up to time t , denoted as $T_{1:t}=\left\{T_{1},T_{2},\ldots ,T_{t}\right\}$ , several metrics have been introduced to assess the degree of continual learning.

Average Accuracy (David Lopez-Paz, 2017), measures the average test accuracy of the model $M_{t}$ on task j after completion of task ${\text{T}}_{\text{t}}$ . It is calculated as shown in Equation 28.


(28) $$\begin{array}{c} Average\;Accuracy\left(M_{t}\right)=\frac{1}{T_{t}}\sum_{j=1}^{T_{t}}acc\left(M_{t},j\right) \end{array}$$

where ${\text{T}}_{\text{t}}$ is the number of tasks completed by the model at time t and $acc\left(M_{t},j\right)$ is the accuracy of the model on the test set after completing task j . The Average Accuracy metric reflects the extent to which the model has mastered the current task after learning task ${\text{T}}_{\text{t}}$ .

### 2.3 Experimental setup

In this study, we compared the performance of several advanced deep learning models as backbone networks for a multi-disease continual learning image classifier. The models used for comparison include:

1. Efficientnet-Lite0 (Tan and Le, 2019), A lightweight convolutional neural network optimized for mobile and edge devices.
2. Regnetx-02 (Radosavovic et al., 2020): A new network design paradigm focusing on parametrizing populations of networks, with the RegNet design space providing simple and fast networks that outperform EfficientNet models while being faster on GPUs.
3. ConvNeXt-S (Liu et al., 2022): A pure ConvNet model family that competes with Transformers in accuracy and scalability for computer vision tasks, achieving high performance on ImageNet classification and outperforming Swin Transformers on various benchmarks.
4. ViT-S/16 (Dosovitskiy et al., 2020): The model we used, which segments the image into fixed-size blocks and processes them using the Transformer architecture.

**Figure 2:**
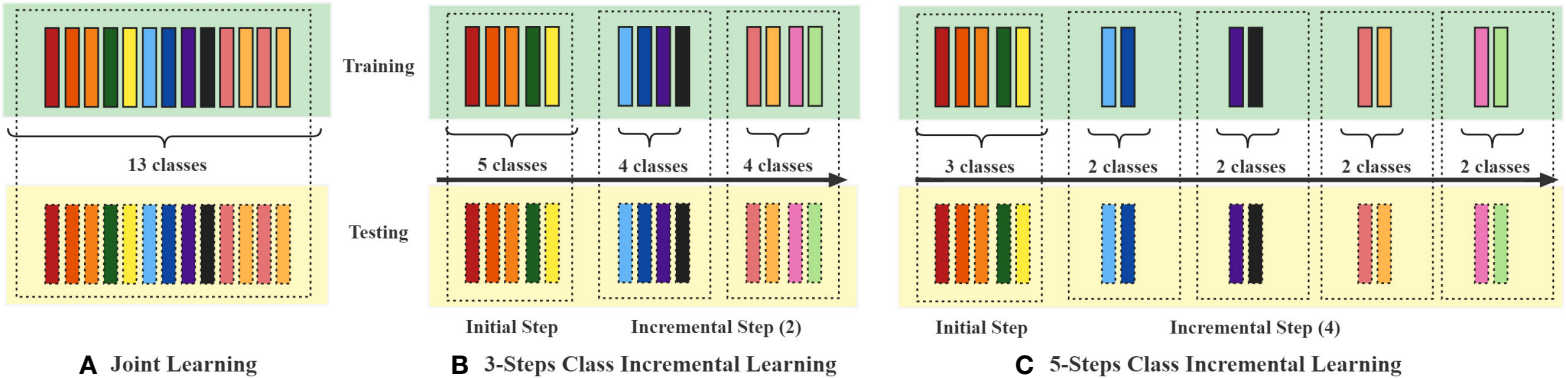
Schematic representation of the experimental setups for Joint learning and 3-steps and 5-steps class-incremental learning. Each coloured block represents a specific crop disease. In **(A)** Joint learning, all categories are trained simultaneously, whereas in the **(B)** 3-steps and **(C)** 5-steps setups, the 13 diseases are divided into 3 and 5 phases for class-incremental learning, respectively.

In evaluating the classification capabilities of the ViT-S/16 model and other models, we conducted joint learning experiments where all image categories were trained simultaneously on the Training set. During the training process, evaluations were carried out on the Validation set, and tests were conducted on the Testing set. In this setup, each model underwent a certain number of iterations, termed “epochs.” In our joint learning experiments, the models were trained for 20 epochs. The training process of the model involved updating its parameters to minimize the discrepancy between the predicted and actual outputs. This procedure employed an optimization algorithm. For our joint learning, we utilized the Adaptive Moment Estimation with Decoupled Weight Decay (AdamW) optimizer, while the AdaMax optimizer was used for incremental learning. The Adam optimizer is a commonly used optimization algorithm in deep learning. Furthermore, we conducted a series of class incremental learning experiments on PlantDiseaseCL using the ViT-S/16 model. In the incremental learning process, the entire dataset was divided into training and testing sets, and segmented into 3-steps and 5-steps learning processes, as shown in **Figure 2**. The model learned the training data of each phase in sequences of 10 epochs and evaluated the Average Accuracy of all learned categories on the testing set after each training step. The experimental design of the 3-steps and 5-steps learning processes assessed our proposed ViT-TV method against other continual learning methods. The considered continual learning methods include:

**Baseline:**

1. Finetuning: An approach where the model is retrained on new data without using any continual learning methods, which may lead to catastrophic forgetting.
2. Freezing: A method that preserve prior task-related knowledge by halting the weight updates of specific layers after the completion of a designated task. In this context, for instance, upon concluding the first task (identified as task index 0), the principal component of the model—often the backbone or lower-level feature extractor—will cease to undergo weight updates. Subsequently, when initiating training for the second task (indexed as task 1), the frozen components will remain unaltered, without any further updates. Nevertheless, the head portion of the model—typically the classifier component—will continue to undergo weight updates to accommodate the requirements of the new task.

**Exemplar Replay Approach:**

1. Class implementing the End-to-end Incremental Learning (EEIL) (Castro et al., 2018): An approach to learn deep neural networks incrementally using new data and a small exemplar set from old classes, integrating distillation measures and cross-entropy loss.
2. Class implementing the Incremental Classifier and Representation Learning (iCaRL) (Rebuffi et al., 2017): a training strategy that enables learning an increasing number of concepts over time from a stream of data in a class-incremental manner. It learns robust classifiers and data representations concurrently, allowing it to gradually acquire knowledge of numerous classes over an extended period, in contrast to alternative strategies that tend to falter quickly.
3. Class implementing the Class Incremental Learning With Dual Memory (IL2M) (Belouadah and Popescu, 2019): A class incremental learning method using fine-tuning and a dual memory system to mitigate catastrophic forgetting, introducing a second memory to store past class statistics.

**Zero-Exemplar Approach:**

1. Learning a Unified Classifier Incrementally via Rebalancing (Lucir) (Hou et al., 2019): A learning method that rebalances the loss function to balance the learning of new and old tasks.
2. Memory Aware Synapses (MAS) (Aljundi et al., 2018): A method that protects prior task knowledge by measuring parameter importance.
3. Synaptic Intelligence (SI) (Zenke et al., 2017): A method that protects prior task knowledge by measuring the importance of each synapse (i.e., connection) in the neural network.
4. Riemannian Walk (RWalk) (Chaudhry et al., 2018): A random walk method used to explore the parameter space and preserve important features.
5. Learning without Forgetting (LwF) (Li and Hoiem, 2017): A method based on the idea of global model function regularization, preserving knowledge through knowledge distillation.
6. Elastic Weight Consolidation (EWC) (Kirkpatrick et al., 2017): A method based on the idea of elastic weight sharing, using the Fisher matrix to store importance parameters for balancing learning between new and old tasks.
7. ViT-TV: Our proposed approach that preserves prior task knowledge by minimizing the TV distance between the attention matrices of new and old tasks, promoting consistent attention regularization.

The algorithmic improvements and assessments are based on the Towards Exemplar-Free Continual Learning in Vision Transformers study (Pelosin et al., 2022), conducted on CIFAR-100 and ImageNet datasets, and benchmarked using the FACIL continual learning evaluation framework (Masana et al., 2023). Experiments were carried out on an NVIDIA V100 GPU utilizing the PyTorch framework, a renowned open-source deep learning platform celebrated for its ease in training and deploying deep learning models.

## 3 Results

### 3.1 Joint training results

#### 3.1.1 Accuracy results

After conducting an analysis of the joint training results for various models, it is evident that there are significant differences in their performance. **Table 2** presents these findings, with each model evaluated based on important metrics such as Precision, Recall, F1-score, and Accuracy, all expressed in percentage terms.

**Table 2:** Comparison of the backbone models of joint training.

Backbone Model	Evaluation Metrics			
	Precision	Recall	F1-score	Accuracy
Efficientnet b0 Lite	0.7623	0.7532	0.7509	0.7544
Regnetx-02	0.9258	0.9221	0.9219	0.9234
ConvNeXt-S	0.9375	0.9361	0.9362	0.9365
ViT-S/16	0.9560	0.9532	0.9528	0.9538

**Figure 3:**
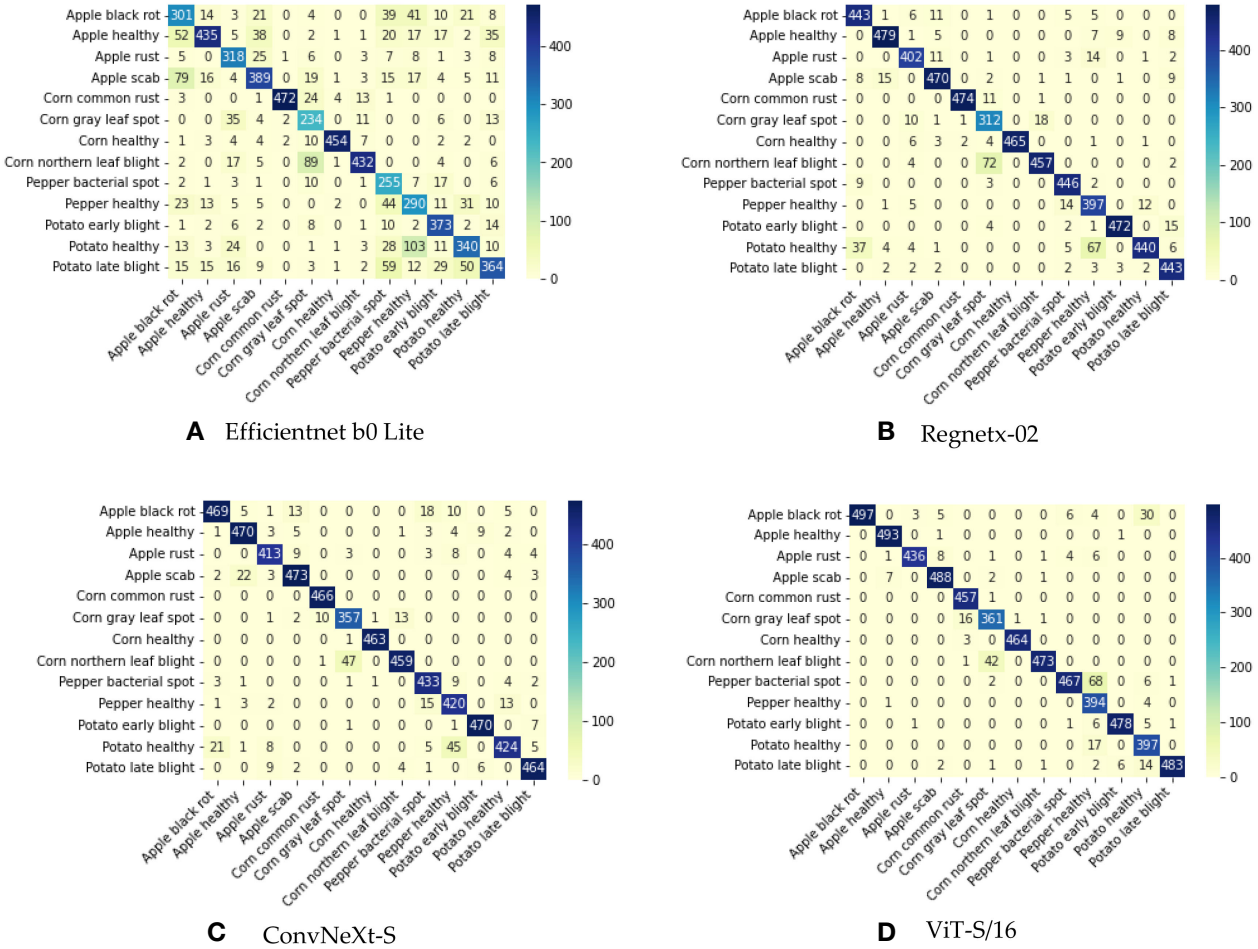
Confusion matrix illustrating the outcomes of joint training. **(A)** Confusion matrix for EfficientNet B0 Lite. **(B)** Confusion matrix for RegNetX-02. **(C)** Confusion matrix for ConvNeXt-S. **(D)** Confusion matrix for ViT-S/16.

The ViT-S/16 model emerges as a paragon of excellence, demonstrating superior performance when juxtaposed with other models. It achieves a precision of 95.60%, a recall of 95.32%, an F1-score of 95.28%, and an accuracy of 95.38%. The foundational model, Efficientnet b0 Lite, lags considerably across all metrics. ViT-S/16 surpasses it by a remarkable margin: 19.37% in precision, 20.00% in recall, 20.19% in F1-score, and 19.94% in accuracy. While Regnetx-02 manages to outdo Efficientnet b0 Lite, it remains in the shadow of ViT-S/16’s prowess. ViT-S/16 outshines Regnetx-02 by 3.02% in precision, 3.11% in recall, 3.09% in F1-score, and 3.04% in accuracy. ConvNeXt-S, despite performing closely to ViT-S/16, still falls short. ViT-S/16 retains a lead with an advantage of 1.85% in precision, 1.71% in recall, 1.66% in F1-score, and 1.73% in accuracy. As further evidenced by the confusion matrix depicted in **Figure 3**, ViT-S/16 exhibits the lowest error rate in recognizing each category.

#### 3.1.2 Various diseases classification results

In the experiment of plant disease classification, the ViT-S/16 model’s prowess, as detailed in **Table 3**, is marked by its precision across diverse leaf species and their respective diseases. For apple leaves, the model excels in distinguishing healthy ones with a precision of 98.22%, a sensitivity of 99.20%, and an F1-score of 98.71%. Black Rot’s detection closely mirrors this performance, albeit slightly lower in precision at 98.01%. Rust and Scab categories exhibit comparable efficacy, with F1-scores of 98.07% and 98.71%, respectively. Corn leaves present an interesting spread: while healthy leaves and Common rust achieved near-perfect scores with F1-scores at 99.79% and 99.37%, the Gray Leaf Spot and Northern Leaf Blight categories recorded 95.62% and 96.89%, respectively. The model’s proficiency extends to pepper leaves, where it identifies healthy leaves with an F1-score of 95.13% and Bacterial Spot at 97.60%. Potato leaves classification emphasizes the model’s capability, especially in the Early Blight category, which stands out with a stellar F1-score of 99.38%.

**Table 3:** Results of ViT-S/16 classification for different diseases.

Leaf Species	Disease Type	Evaluation Metrics		
		Precision	Sensitivity	F1-score
Apple	Healthy	0.9822	0.9920	0.9871
	Black Rot	0.9801	0.9920	0.9860
	Rust	0.9818	0.9795	0.9807
	Scab	0.9842	0.9901	0.9871
Corn	Healthy	0.9957	1.0000	0.9979
	Gray Leaf Spot	0.9287	0.9854	0.9562
	Common rust	0.9979	0.9895	0.9937
	Northern Leaf Blight	0.9934	0.9455	0.9689
Pepper	Healthy	0.9392	0.9638	0.9513
	Bacterial Spot	0.9730	0.9791	0.9760
Potato	Healthy	0.9815	0.9320	0.9561
	Early Bight	1.0000	0.9876	0.9938
	Late Blight	0.9814	0.9814	0.9814

Deep learning models inherently have the capability to autonomously distill representative features from images. The caliber of these extracted features fundamentally influences the ensuing classification performance. To rigorously assess the feature quality, we procured models from three predominant deep learning image classification paradigms: CNN and ViT. We extracted the penultimate feature vectors by tapping into the last layer of each model’s feature extractor, producing multidimensional vectors. These vectors were subsequently projected onto a two-dimensional plane employing the t-SNE dimensionality reduction technique (Van Der Maaten and Hinton, 2008).

**Figure 4:**
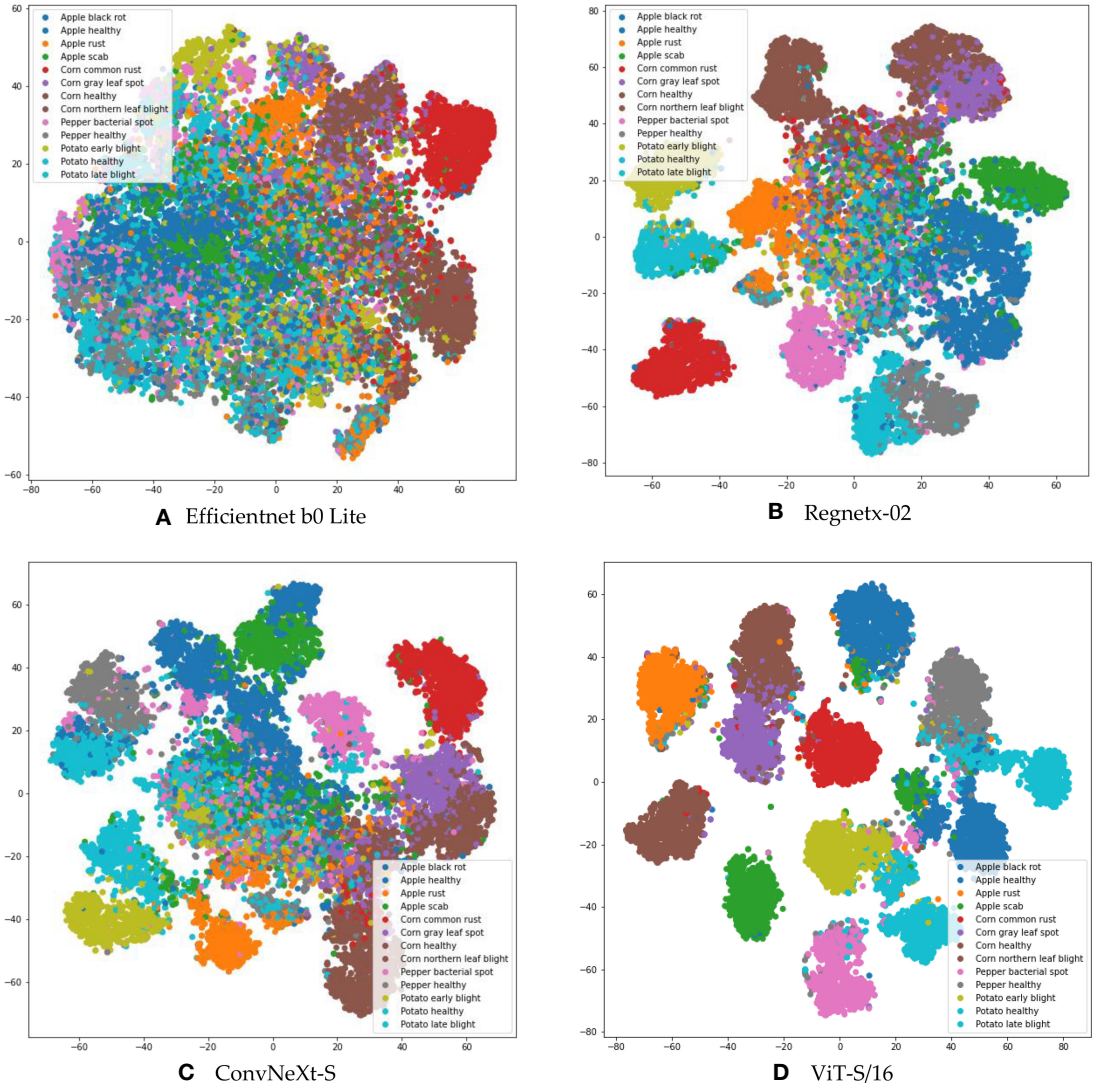
Feature space visualization of various models, depicting **(A)** t-SNE analysis for EfficientNet B0 Lite, **(B)** t-SNE analysis for RegNetX-02, **(C)** t-SNE analysis for ConvNeXt-S, and **(D)** t-SNE analysis for ViT-S/16.

**Figure 4** graphically represents the t-SNE outcomes for various models, with distinct colorations symbolizing different disease categories. Analyzing these t-SNE feature distribution plots proffers enlightening conclusions. The scatter plots derived from the Efficientnet b0 Lite, Regnetx-02, and ConvNeXt-S models manifest an overlap, delineating an absence of discernible boundaries between different classes. Such intertwined high-dimensional features potentially complicate the task for subsequent classifiers, leading to subpar classification accuracy.

In contrast, the feature distribution from ViT-S/16 stands out. There’s a clearer distinction between different classes of features. This striking separation highlights the ability of ViT-S/16 not only to reduce intra-class variability, but also to effectively separate feature embeddings.

### 3.2 Continual learning results

#### 3.2.1 Average accuracy

In our continual learning research, we compared the average accuracy of class-incremental learning based on the ViT-S/16 model on the PlantDiseaseCL dataset under various strategies, as shown in **Table 4**. For baseline strategies, we explored two primary methods:

**Table 4:** Average accuracy results for class-incremental learning on PlantDiseaseCL (Based on ViT-S/16).

Strategy	Methods	Examples	Average Accuracy	
			3-Steps	5-Steps
Baseline	Freezing	0	0.3301	0.2000
	Finetuning	0	0.4531	0.3799
Exemplar Replay Approach	EEIL	20	0.5649	0.3479
	IL2M	20	0.6058	0.4990
	ICaRL	20	0.6488	0.5216
Zero-Exemplar Approach	LUCIR	0	0.3323	0.1998
	SI	0	0.4557	0.4130
	MAS	0	0.4658	0.3990
	EWC	0	0.4741	0.4108
	RWalk	0	0.4819	0.3961
	LwF	0	0.5889	0.3143
	TV(ours)	0	0.7077	0.5661

1. Fine-tuning, which eschews any continual learning techniques and solely relies on the original ViT model for continual learning. This approach achieved average accuracies of 0.4531 and 0.3799 for 3-Steps and 5-Steps learning, respectively.
2. The freezing strategy, which exhibited slightly inferior performance, with average accuracies of 0.3301 and 0.2000, respectively.

When employing the Exemplar Replay Approach, the system can retain a certain number of samples for subsequent learning. In our experiments, the maximum number of stored samples for all these methods was set at 20. Among them, ICaRL led the pack with scores of 0.6488 and 0.5216, marking an improvement of 8.39% and 4.3% over its counterparts, EEIL and IL2M strategies, respectively.

However, the most salient results were observed under our proposed Zero-Exemplar Approach utilizing the TV method. Remarkably, despite not necessitating the storage of any exemplar samples, this approach achieved average accuracies of 0.7077 and 0.5661 for 3-Steps and 5-Steps learning, respectively. Not only did this significantly outperform other strategies that don’t employ exemplar replay (compared to LUCIR, the TV strategy improved by 37.54% and 36.63% for 3-Steps and 5-Steps, respectively; and when juxtaposed with SI, MAS, EWC, and LwF strategies, the gains were 25.20%, 24.19%, 23.66%, and 11.88% for 3-Steps, and 15.31%, 16.71%, 15.53%, and 25.18% for 5-Steps, respectively), but more notably, the TV strategy, even without using exemplar samples, outperformed some strategies that did. For instance, compared to ICaRL, the TV strategy improved by 5.89% in 3-Steps learning. This is a significant finding as, conventionally, strategies employing exemplar samples in class-incremental learning tend to exhibit superior continual learning performance.

#### 3.2.2 Incremental learning processes results

In the realm of continual learning, ensuring consistent performance improvement during the incremental learning phase stands as one of the foremost challenges, especially when evaluating against diverse benchmarks. To delve deeper into this process, we employ the ViT-S/16 model and present the evolution of class-incremental learning performance on the PlantDiseaseCL dataset.

**Figure 5:**
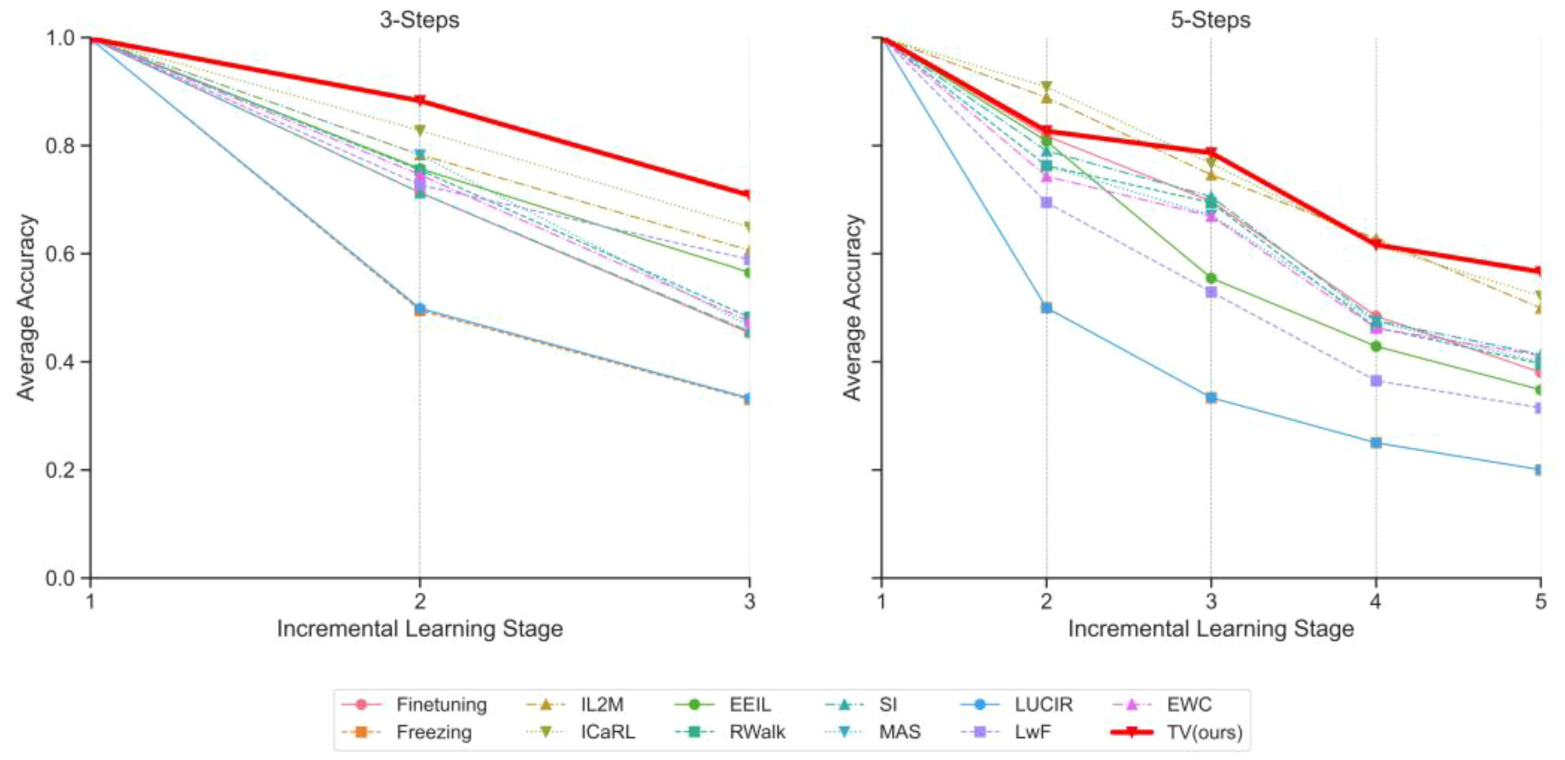
Class-incremental learning performance evolution on PlantDiseaseCL: Reported Top-1 average accuracy after each stage learning. Left figure shows evaluation with 3 steps, while the right figure shows evaluation with 5 steps (Based on ViT-S/16).

**Figure 5** reveals subtle distinctions among various continual learning strategies during the 3-Steps and 5-Steps learning phases. The left panel represents the 3-Steps evaluation, unveiling pronounced disparities in strategy effectiveness. Likewise, the right panel encapsulates a broader 5-Steps progression, reinforcing these observations.

Upon a more thorough analysis of these metrics, although most strategies exhibit near-identical accuracy levels in the initial phase, the trajectory diverges thereafter. Approaches such as Freezing and LUCIR appear to respond inadequately to the challenges of continual learning, with accuracy sharply declining as steps progress. Conversely, strategies like IL2M and ICaRL manifest a more gradual decline. However, even within these methods, the rate of degradation varies.

Next, we turn to our proposed strategy, TV (ours). Notably, it not only maintains its momentum but can be argued to exhibit the slowest rate of average accuracy decline at each stage. As stages progress, TV (ours) consistently excels in retaining knowledge and adapting to new information. In the 3-Steps scenario, it achieves an admirable accuracy of 0.7077 in the third stage, surpassing its closest competitor by a substantial margin. In the 5-Steps evaluation, the TV approach similarly takes the lead, achieving the highest final average accuracy score of 0.5661.

In summary, our TV strategy demonstrates exceptional performance across stages. Its prowess is particularly evident in the achieved final average accuracy scores, outperforming competitors significantly in both 3-Steps and 5-Steps evaluations.

#### 3.2.3 Comparative experiments on variants of multiple attention alignment methods

In the Continual Learning segment, assessing the efficacies of various attention alignment methods, especially under consistent ViT conditions, is of paramount importance. The results shed light on how different attention alignment techniques, when based on diverse distance metrics, influence the overall learning performance as shown in **Table 5**. Interestingly, all variants of attention alignment methods commence their journey from a nearly similar starting point, but the divergence becomes evident in subsequent stages. A notable observation is the performance of the ‘ Original ViT’. Despite being the foundational model, its average accuracy is only 0.4531 and 0.3799 for 3-Steps and 5-Steps respectively, which points towards the significance of integrating additional distance metrics for enhanced performance.

**Table 5:** Comparative results on variants of multiple attention alignment methods.

Methods	3-Steps Average Accuracy	5-Steps Average Accuracy
Original ViT	0.4531	0.3799
ViT + JS Divergence	0.6106	0.5280
ViT + Hellinger Distance	0.3328	0.2000
ViT + Bhattacharyya Distance	0.6136	0.5427
ViT+TV Distance	0.7077	0.5661

Notably, methods employing ‘JS Divergence’ and ‘Bhattacharyya Distance’ exhibited significant improvements. The principle behind the ‘JS Divergence’ method is rooted in the Jensen-Shannon divergence metric. For two given probability distributions, P and Q, it first calculates their relative entropy with respect to their average distribution, yielding a measure of divergence for each distribution from the average. The average of these two relative entropies is then taken as the Jensen-Shannon divergence, serving as a measure of the difference between P and Q.

The ‘Bhattacharyya Distance’, on the other hand, is a metric designed to gauge the similarity between two probability distributions. It involves taking the square root of each element of the two distributions, multiplying them pairwise, and then summing up all the products. The negative logarithm of this sum is then taken. This value, which essentially represents the cross-entropy between the two distributions, quantifies the amount of information shared between them. A smaller Bhattacharyya Distance indicates greater similarity between the distributions, and vice versa. Our experimental results underscored the efficacy of both the JS Divergence and Bhattacharyya Distance methods. Particularly, the ‘Bhattacharyya Distance’ method manifested a significant accuracy enhancement of 16.05% and 16.28%.

Furthermore, in the ‘ViT + Hellinger Distance’ method, we utilized the Hellinger distance, which measures the similarity between two probability distributions by calculating the Euclidean distance of their square roots. During computation, we introduced a normalization factor of $\left(\frac{1}{\sqrt{2}}\right)$ . Surprisingly, this method exhibited a declining trend in performance across two distinct steps, registering drops of 12.03% and 17.99% respectively when compared to the Original ViT. This suggests that not all attention alignment techniques universally yield positive outcomes in such contexts.

However, the true standout is our proposed ‘ViT+TV Distance’ method. Demonstrating consistent superiority over other techniques, it achieved an average accuracy of 0.7077 for 3-Steps and an impressive 0.5661 for 5-Steps. These figures not only highlight the robustness and supremacy of the TV Distance in attention alignment but also accentuate its potential in striking an optimal balance between accuracy and adaptability in continual learning environments.

## 4 Discussion

In this study, we introduce a novel mathematical paradigm for continual learning in the domain of crop disease defect recognition. By proposing the innovative ViT-TV framework, we further amplify our contribution, addressing the challenges of multi-disease image recognition in crops within the ViT architecture. We employ the Total Variation distance loss (TV-Loss) to quantify the disparity between current and prior attention distributions, fostering attention consistency and mitigating the catastrophic forgetting inherent to ViT in the absence of prior task samples. With this new framework, we offer a solution for continual learning in intricate scenarios like crop disease recognition.

Distinctively, the ViT-TV method bridges the gap between stability and plasticity in model learning. By incorporating TV-Loss into its internal architecture and co-optimizing TV-Loss with cross-entropy loss, it ensures attention consistency when assimilating new tasks, allowing the model to adapt and learn without significantly compromising previously acquired knowledge. Retaining historical knowledge is paramount for accurate and reliable disease recognition in crops, marking a significant stride forward.

Compared to established Zero-Exemplar Approach types of continual learning techniques: SI focuses on safeguarding synaptic weights to alleviate catastrophic forgetting, EWC protects vital knowledge by regularizing the network’s global weights, and LwF relies on knowledge transfer techniques from the theory of knowledge distillation, ViT-TV stands out by addressing attention consistency. Maintaining attention consistency becomes crucial in the domain of food and crop disease image recognition, especially when confronted with subtle variations in different disease manifestations. The ViT-TV framework, grounded on TV distance and attention alignment, offers superior average accuracy metrics in 3-step and 5-step class incremental learning experiments on PlantDiseaseCL by holistically considering attention consistency, stability, and knowledge preservation, presenting a theoretically robust and practically effective approach to maintaining model stability when recognizing multiple diseases.

Further juxtaposing the ViT-TV framework with exemplar replay methods (e.g., ICaRL) accentuates the superiority of our approach. While ICaRL adeptly uses exemplar samples to combat forgetting, the ViT-TV framework obviates the need for sample storage. The philosophy underpinning our method posits that attention consistency based on Total Variation distance plays a pivotal role in memory retention and transfer across tasks. Unlike methods predominantly reliant on archiving exemplar samples to counteract forgetting, ViT-TV captures the attention distribution of prior tasks, amalgamating it with the attention from new learning, effectively mitigating the risk of catastrophic forgetting.

In this research, we also delve deeper into how different distance metrics can be employed to regularize attention maps, optimizing model performance. The intrinsic value of attention mechanisms lies in enabling the model to focus on pivotal parts of the input, thereby capturing salient information. However, these focal points may vary with task or model iterations. Thus, selecting an apt distance metric to accentuate or diminish these differences is crucial. Integrating the TV distance into the ViT’s attention mechanism offers a potent strategy for addressing the continual learning recognition challenges of multiple diseases in food and crops.

## 5 Conclusions

In summary, our ViT-TV framework establishes a pioneering approach to address the continual learning challenges in the domain of crop disease defect recognition. By adeptly leveraging attention consistency and the Total Variation distance loss, our method contributes to the intelligent evolution of the agricultural industry, ensuring that AI models possess sustainable growth and augmented disease recognition capabilities.

## Data availability statement

The original contributions presented in the study are included in the article/supplementary material. Further inquiries can be directed to the corresponding author.

## Author contributions

BW: Methodology, Writing – original draft, Writing – review & editing.
